# Unique Bone Marrow Findings of FDG-PET/CT in Acute Leukemia in Children: Comparison to Inflammatory Diseases

**DOI:** 10.3390/children12091218

**Published:** 2025-09-11

**Authors:** Yuta Suenaga, Kazuo Kubota, Motohiro Matsui, Atsushi Makimoto, Junko Yamanaka, Shinji Mochizuki, Masatoshi Hotta, Miyako Morooka Chikanishi, Hiroyuki Shichino

**Affiliations:** 1 Department of Pediatrics, Japan Institute for Health Security National Center for Global Health and Medicine, 1-21-1 Toyama, Shinjuku-ku 162-8655, Japan; yuta.gp3@gmail.com (Y.S.); yamanaka.j@jihs.go.jp (J.Y.); mochizuki.s@jihs.go.jp (S.M.); shichino.h@jihs.go.jp (H.S.); 2 Division of Critical Care Medicine, Saitama Children’s Medical Center, 1-2 Shintoshin, Chuo-ku, Saitama 330-8777, Japan; 3 Department of Radiology, Southern TOHOKU General Hospital, 7-115 Yatsuyamada, Koriyama-shi 963-8563, Japan; kkubota@cpost.plala.or.jp; 4 Department of Hematology and Oncology, Tokyo Metropolitan Children’s Medical Center, 2-8-29 Musashidai, Fuchu 183-8561, Japan; atsushi_makimoto@tmhp.jp; 5 Division of Molecular Epidemiology, Jikei University School of Medicine, Tokyo 105-8461, Japan; 6 Department of Laboratory Medicine, Tokyo Metropolitan Children’s Medical Center, Tokyo 183-8561, Japan; 7 Department of Nuclear Medicine, Japan Institute for Health Security National Center for Global Health and Medicine, 1-21-1 Toyama, Shinjuku-ku 162-8655, Japan; hotta.m@jihs.go.jp; 8 Department of Neuroradiology, Tokyo Metropolitan Neurological Hospital, 2-6-1 Musashidai, Fuchu 183-0042, Japan; miyachan777@hotmail.com

**Keywords:** Fluorine-18 fluorodeoxyglucose positron emission tomography/computed tomography (FDG-PET/CT), acute leukemia, bone marrow

## Abstract

**Background/Objectives:** Fluorine-18 fluorodeoxyglucose positron emission tomography/computed tomography (FDG-PET/CT) is a valuable imaging modality for detecting malignancies and diagnosing fever of unknown origin (FUO). However, data regarding FDG accumulation in bone marrow among pediatric acute leukemia (AL) cases are limited. In this study, we aimed to compare FDG-PET/CT findings between children with AL and those with inflammatory diseases (IDs), including FUO, and develop a scoring system for differential diagnoses. **Methods:** We retrospectively analyzed FDG-PET/CT findings in six children with AL and 22 with IDs. The maximum standardized uptake value (SUV max), visual score (VS), and spread score (SS) were evaluated across various bone marrow sites, including vertebrae, pelvic bone, humerus, forearm, and femur. Statistical analysis consisted of Mann–Whitney U test for group comparisons and receiver operating characteristic curve (ROC)/area under the curve (AUC) analyses to assess diagnostic performance. **Results:** SUV max, VS, and SS were significantly higher in children with AL across all evaluated sites. The combined VS + SS scoring system yielded the highest diagnostic accuracy. A simplified version using only the VS of the middle humerus and femur plus the SS showed comparable effectiveness. **Conclusions:** FDG-PET/CT in children with AL showed high FDG accumulation in bone marrow areas in the whole body. The simple scoring system, which comprises FDG accumulation in the middle portion of the extremities and the whole body, appears to be helpful in distinguishing AL from IDs in children. FDG-PET/CT-based visual scoring may provide supportive information alongside conventional diagnostics in pediatric acute leukemia.

## 1. Introduction

Acute leukemia (AL) is the most common malignancy in children [1]. In Japan, approximately 600 to 700 new cases are diagnosed annually, with an estimated incidence of 4 to 5 per 100,000 children [2]. Diagnostic confirmation typically relies on the morphological evaluation of malignant cells obtained via bone marrow aspiration or biopsy. Nevertheless, when pathological evaluation is inconclusive or limited, additional diagnostic measures may help support clinical decision-making. Fluorine-18 fluorodeoxyglucose positron emission tomography combined with computed tomography (FDG-PET/CT) is widely used for diagnosis, staging, treatment monitoring, and relapse detection across a range of malignancies. While the clinical application of bone marrow FDG uptake patterns in AL has primarily been limited to detecting extramedullary involvement in acute myeloid leukemia [3], the authors of a few small studies have suggested that it may assist in the differential diagnosis of AL [4,5]. In contrast, FDG-PET/CT has also proven useful in evaluating pediatric patients with fever of unknown origin (FUO) [6], and high bone marrow FDG uptake has been reported in various inflammatory diseases (IDs), including FUO [7,8,9]. However, to date, there are no studies in which bone marrow FDG uptake has been directly compared between pediatric AL and IDs. In this study, we aimed to establish a simple visual scoring system to differentiate between children with AL and those with IDs by comparing FDG-PET/CT findings between the two groups.

## 2. Materials and Methods

### 2.1. Study Design and Patients

This single-center retrospective study reviewed the medical records of patients admitted to the Department of Pediatrics at National Center for Global Health and Medicine (NCGM) between January 2011 and December 2015. This study was approved by the Institutional Review Board of the NCGM (approval number: NCGM-G-002526-00) and was conducted in accordance with the tenets of the Declaration of Helsinki. Informed consent was not required, as only fully anonymized data were used. An information leaflet was posted in the hospital to inform patients and their guardians about the general use of clinical data for research purposes. We selected a total of 67 patients under the age of 18 who underwent FDG-PET/CT in the study period. A total of 28 patients (6 AL and 22 ID individuals) out of 67 were considered eligible for the analysis after the exclusion of cases presenting the following: (1) pediatric cancers without AL (*n* = 32), (2) relapse or after treatment (*n* = 6), (3) low accumulation of FDG in the brain (*n* = 1). The six AL patients comprised three with acute lymphoblastic leukemia and three with acute myelogenous leukemia. All were previously untreated. Of the 22 ID children, seven presented with necrotizing lymphadenopathy, two with reactive lymphadenitis, two with fever of unknown origin (one of which presenting complications of IgA vasculitis), two with purulent lymphadenitis, two with sinusitis (one of which presenting complications of immune thrombocytopenia), and seven with other inflammatory diseases (viral lymphadenitis, upper respiratory inflammation with cervical mass and growth hormone deficiency, mycoplasma pneumoniae, lung abscess, exanthema subitum with encephalopathy, periodic fever, and tonsillitis).

### 2.2. FDG PET/CT Scanning

FDG-PET/CT images were obtained using Biograph mCT (Siemens, Erlangen, Germany) and Discovery PET/CT 600 (General Electric Healthcare, Waukesha, WI, USA). The patients were instructed to fast for at least six hours, and the blood glucose level was monitored using a glucometer (Asetsense Duo, HORIBA MEDICAL, Kyoto, Japan) prior to injection. Approximately 60 min after the intravenous injection of 18F-FDG according to the guideline [10], whole-body PET/CT scanning was performed. The scanning acquisition range was from the head to the mid-thigh or toe. CT without contrast enhancement was performed as a spiral scan with the following settings: 120 kVp, 0.5 s rotation time, 5 mm slice thickness, and low-dose tube current modulation (the Noise Index and quality reference mAs were SD: 25 and 150 mAs, respectively). A PET scan was acquired in 3-dimensional mode for 2 to 3 min per bed covering 4 to 11 bed positions.

### 2.3. Evaluation of FDG-PET/CT Imaging

**Figure 1:**
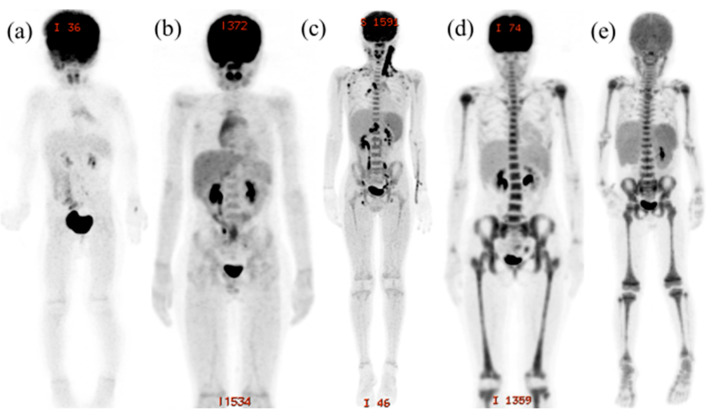
MIP images. The VS classifies degree of FDG accumulation in bone marrow areas compared with the liver into 4 grades. Analysis example focusing on the pelvis: (**a**) A 3-year-old girl with sinusitis as a complication of immune thrombocytopenia showing no FDG accumulation in pelvic bone marrow; the score was 0. (**b**) An 11-year-old boy with reactive lymphadenitis with lower FDG accumulation in pelvic bone marrow than in the liver; the score was 1. (**c**) An 11-year-old boy with necrotizing lymphadenopathy showing the same FDG accumulation in both pelvic bone marrow and the liver; the score was 2. (**d**) A 17-year-old girl with acute myelogenous leukemia with stronger FDG accumulation in pelvic bone marrow than in the liver; the score was 3. The SS classifies the spread of FDG accumulation in bone marrow areas into 2 grades. (**e**) A 5-year-old boy with acute lymphoblastic leukemia with clear FDG accumulation in the vertebrae, pelvic bone marrow, the humerus, and the femur; the score was 4.

The evaluation of FDG-PET/CT imaging, based on maximum intensity projection (MIP) images, was performed in 11 body areas per patient (i.e., vertebrae, pelvic bone, humerus (proximal, mid, and distal), forearm (proximal, mid, and distal), and femur (proximal, mid, and distal)). We measured the maximum standardized uptake value (SUV max) in the bone marrow of the 11 areas in order to make a quantitative comparison between the two groups. We also evaluated the visual score (VS), which classifies the degree of FDG accumulation compared with the liver into 4 grades (0: no accumulation; 1: lower accumulation; 2: equivalent accumulation; 3: greater accumulation) (Figure 1), and the spread score (SS), which classifies the spread of FDG accumulation in bone marrow areas into 2 grades (0: partial accumulation; 1: whole accumulation) (Figure 1). The VS was evaluated in the bone marrow of the 11 areas, while the SS was evaluated in 5 anatomic sites: the vertebrae, the pelvic bone, the humerus, the forearm, and the femur. The scoring was independently evaluated by two pediatricians (Y.S, M.M.) based on the previous literature [11], and confirmed by radiologists (M.M.C., K.K.). In case of discrepancies, a consensus meeting was held to reach agreement. The interobserver reliability between the two pediatricians was quantified using Cohen’s kappa statistic, showing excellent agreement (κ = 0.862 for both VS and SS). The decision criteria for the SUV max and the VS are as follows: (1) take the higher value between the right and left scores, (2) take the average of the top 3 scores in the vertebrae, and (3) take the average of the bilateral iliac crest. For the SS, the criteria are as follows: (1) identify all vertebrae from the cervical to the lumbar regions, (2) identify the whole pelvic bone (sacrum, ilium, ischium, and pubis), and (3) identify the whole long bone from the proximal epiphysis to the distal epiphysis.

### 2.4. Statistics

PASS 11 was used for determining the power of the study based on the sample size. The power was 0.85, assuming a level of significance of 0.05; the logarithms of the VS and odds ratio were 0.5, and we considered 6 cases and 22 controls. R version 4.2.2 (R Core Team, Vienna, Austria) and Python 3.10 (Python Software Foundation, Wilmington, DE, USA) were used for statistical analyses. We compared the blood tests and the data (SUV max, VS, and SS) for each bone marrow area between the AL and ID groups by using the Mann–Whitney U-test. All *p*-values were two-sided and considered significant at *p* < 0.05. The interobserver reliability of VS and SS was evaluated using Cohen’s kappa statistic. A scoring system for differential diagnoses between AL and IDs was made with receiver operating characteristic (ROC) and area under the curve (AUC) analyses.

## 3. Results

The clinical characteristics of the blood tests between the AL and ID groups are shown in Table 1 and Table S1. Differences in median age and sex were not statistically significant between the AL and ID groups. Hemoglobin (Hb) and Platelet (Plt) in the AL group were significantly lower than in the ID group, reflecting pancytopenia in AL There were also no differences in White blood cell (WBC) count, C-reactive protein (CRP), ferritin, soluble interleukin-2 receptor (sIL-2R), and erythrocyte sedimentation rate (ESR).

**Figure 2:**
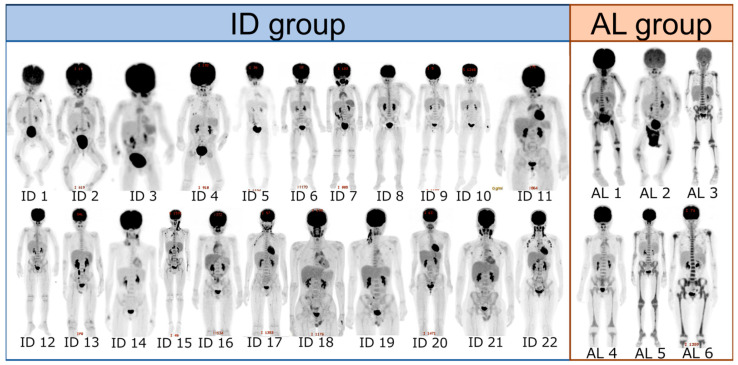
Displays maximum intensity projection (MIP) images for each case. Each MIP image is labeled with the corresponding patient number below.

**Figure 3:**
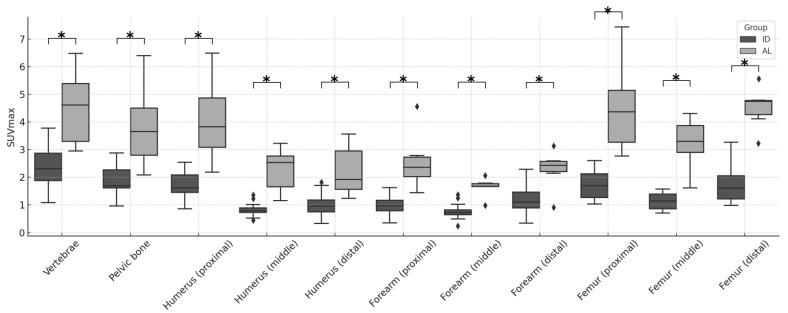
Box plots showing comparisons of SUV max between AL and ID groups in 11 body areas (i.e., vertebrae, pelvic bone, humerus (proximal, mid, and distal), forearm (proximal, mid, and distal), and femur (proximal, mid, and distal)). * indicates a *p*-value < 0.01.

**Figure 4:**
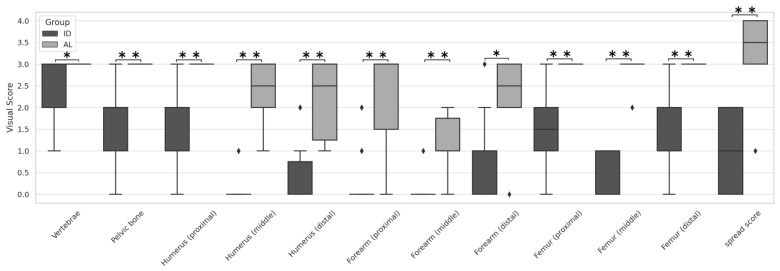
Box plots showing comparisons of visual score between the AL and ID groups in 11 body areas (i.e., vertebrae, pelvic bone, humerus (proximal, mid, and distal), forearm (proximal, mid, and distal), and femur (proximal, mid, and distal)). * indicates *p*-value < 0.05. ** indicates *p*-value < 0.01.

**Figure 5:**
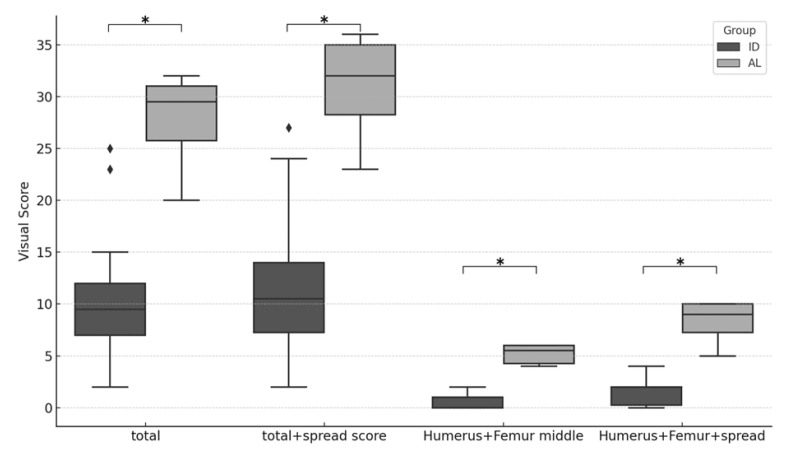
Box plots comparing total visual score and mid-humerus/femur visual score, with and without inclusion of the spread score, between the AL and ID groups. * indicates *p*-value < 0.01.

MIP images of all 28 patients—6 with AL and 22 with ID—are presented for evaluation in Figure 2. The SUV max in each of the 11 areas in the two groups was significantly higher in AL, as shown in Figure 3 and Table S2. Similarly, the VS and the SS were significantly higher in AL in all areas, as shown in Figure 4, Figure 5 and Table S3, respectively. The Kappa statistic (Cohen) was 0.862 in the VS and the SS. We found that the SUV max and the VS of the proximal and distal sections of the extremities (humerus, forearm, and femur) tended to be higher than those of the middle sections in both groups. Thus, the application of the VS in the middle portion of the extremities appears useful in distinguishing AL from IDs.

**Figure 6:**
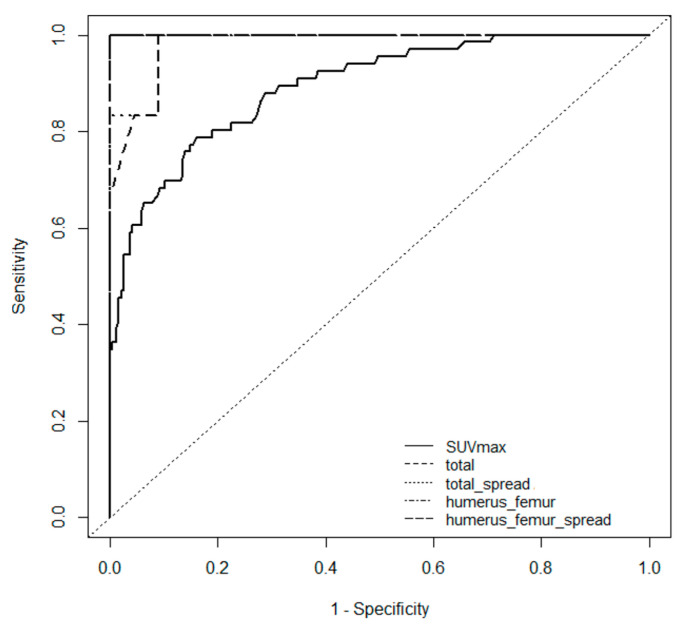
ROC curves between the AL and ID groups for each scoring system: SUV max, total visual score (VS), total VS + spread score (SS), mid-humerus/femur VS and mid-humerus/femur VS + SS. Long-dash line: mid-humerus/femur VS ROC curve (cutoff, 4; sensitivity, 1.00; specificity, 1.00; AUC, 1.00). Dot-dash line: mid-humerus/femur VS ROC curve (cutoff, 3; sensitivity, 1.00; specificity, 1.00; AUC, 1.00). Dotted line: total VS + SS ROC curve (cutoff, 20; sensitivity, 1.00; specificity, 0.91; AUC, 0.985). Dashed line: total VS ROC curve (cutoff, 18; sensitivity, 1.00; specificity, 0.91; AUC, 0.981). Solid line: SUV max ROC curve (cutoff, 2.06; sensitivity, 0.79; specificity, 0.84; AUC, 0.892).

Figure 6 shows the ROC curves and AUCs between the AL and ID groups for each scoring system, i.e., SUV max, VS, and VS + SS. The results show that VS + SS was the most useful system for distinguishing AL from IDs in children (SUV max: cutoff, 2.06; sensitivity, 0.79; specificity, 0.84; AUC, 0.892. VS: cutoff, 18; sensitivity, 1.00; specificity, 0.91; AUC, 0.981. VS + SS: cutoff, 20; sensitivity, 1.0; specificity, 0.91; AUC, 0.985). In addition, we examined the ease of use of the scoring systems for distinguishing AL from IDs by comparing ROC curves and AUCs in all combinations of the VS and the SS. We found that the VS of the middle portion of humerus and femur combined with the SS showed the same results (cutoff, 4; sensitivity, 1.00; specificity, 1.00; AUC: 1.00) as VS + SS. This shows that the VS of the middle portion of humerus and femur combined with the SS has better efficacy in distinguishing AL from IDs as VS + SS.

## 4. Discussion

In this study, we demonstrated that our scoring system based on FDG-PET/CT findings is useful in differentiating AL from IDs in children. We found that the SUV max, the VS, and the SS were significantly higher in the bone marrow of patients with AL compared with those with IDs, despite the presence of inflammation according to blood tests. Notably, the VS in the mid-diaphyseal region of the extremities was particularly useful in distinguishing AL from IDs. These results suggest that increased FDG uptake in the mid-portions of the humerus and the femur, along with widespread bone marrow involvement, represents a characteristic FDG-PET/CT finding in pediatric acute leukemia.

Our study also showed clear differences in bone marrow FDG uptake between the AL and ID groups on PET/CT. It is well known that high FDG accumulation in bone marrow in AL reflects increased number and metabolic activity of leukemic cells [5]. However, few authors have thoroughly investigated the specific FDG uptake patterns in pediatric AL. In contrast, FDG accumulation may also be elevated in IDs [7,8,9] due to inflammatory reactions within bone marrow. In our study, the SUV max, the VS, and the SS across all 11 evaluated bone marrow regions were significantly higher in children with AL than in those with IDs. Additionally, FDG uptake in the mid-diaphyseal regions of the extremities tended to be higher in the AL group than in the ID group. These findings support the utility of our scoring system for differentiating AL from IDs and indicate that increased intensity and broader distribution of FDG uptake are characteristic features of pediatric AL on FDG-PET/CT.

We further demonstrated that FDG-PET/CT in pediatric AL reveals both high intensity and wide distribution of FDG uptake in bone marrow, as evaluated using the VS and the SS. In all 11 defined bone marrow regions per patient, the SUV max, the VS, and the SS were consistently higher in the AL group than in the ID group. The authors of previous studies have reported high FDG uptake in the vertebrae, pelvic bones, and other skeletal sites in AL and have suggested that AL should be considered in the differential diagnosis when diffuse marrow uptake is present [5]. Li et al. [11] showed that the SUV max is a useful marker for quantifying metabolic activity and may be a prognostic factor in malignancy. However, the SUV max reflects only the most active voxel and does not account for total lesion burden. Metabolic tumor volume (MTV) and total lesion glycolysis (TLG), calculated by multiplying MTV by the mean SUV, have been introduced as more comprehensive indicators of tumor metabolism [12]. Based on these concepts, we aimed to evaluate both the intensity and extent of FDG uptake in bone marrow by using simple visual assessment tools. Visual scoring methods have previously been used to compare FDG uptake across organs, often using liver activity as a reference [13,14]. Referring to these methods, we established the VS and the SS. In this study, these scores were significantly higher in children with AL in all bone marrow regions, similar to the SUV max. Given that the VS and the SS are easier to apply in clinical practice than the SUV max or MTV/TLG, they may serve as useful surrogate measures for evaluating diffuse bone marrow involvement in pediatric AL.

Our findings are consistent with those reported by Zhang et al., who analyzed FDG-PET/CT in 134 adult patients with diffusely increased bone marrow uptake. They found that intense FDG accumulation in the humerus and femur was significantly associated with bone marrow malignant infiltration (BMI) and identified humeral uptake over two-thirds and a high femoral SUV max as independent predictors of malignancy, obtaining an AUC of 0.918 when combining these metrics with age and neutrophil count [15]. In contrast, our study focused specifically on pediatric patients with acute leukemia and included a detailed anatomical evaluation of 11 bone marrow regions using both the VS and the SS. This allowed us to characterize both the intensity and distribution of FDG uptake in children, providing a practical and pediatric-specific approach to differentiating AL from infection.

Our study also showed that FDG accumulation in the proximal and distal parts of the extremities tended to be higher than in the mid-portions in both groups. We speculate that this result may reflect physiological FDG accumulation in red marrow and growth plate cartilage. Previous studies have shown physiological FDG uptake in bone marrow and related structures on PET/CT [16]. Bone marrow is composed of red and yellow marrow. Tanaka et al. [17] reported that red marrow has hematopoietic function, while yellow marrow does not. Vogler JB 3rd et al. [18] showed that the vertebrae, pelvis, sternum, ribs, scapulae, skull, and proximal femur and humerus retain red marrow throughout life. However, most long bones of the extremities contain red marrow at birth, which gradually converts into yellow marrow with age. For example, red-to-yellow marrow conversion in the femur begins in the diaphysis (ages 1–10), proceeds to the distal metaphysis (ages 10–20), and completes by around 24 years [19]. This transformation progresses from peripheral to central, and the last parts to convert are the proximal metaphyses of the femur and humerus [20]. Growth plate cartilage also contributes to endochondral ossification through chondrocyte proliferation and mineralization [21]. These physiological changes should be taken into account when interpreting FDG uptake in pediatric extremities.

We developed a scoring system for differentiating AL from IDs based on FDG-PET/CT findings. Among the evaluated metrics—SUV max, VS, and the combination VS + SS—the latter showed the highest diagnostic performance according to ROC analysis, with a cutoff of 20, a sensitivity of 1.00, a specificity of 0.91, and an AUC of 0.985. We also evaluated a simplified version using the VS of the mid-diaphysis of humerus and femur combined with the SS, which achieved better diagnostic performance (cutoff, 4; sensitivity, 1.00; specificity, 1.00; AUC, 1.00). These results indicate that the simplified model may offer a practical alternative for clinical use.

To our knowledge, no authors have previously used visual assessment alone to differentiate AL from IDs in pediatric patients. Our visual scoring system offers a simple, re-producible, and pediatric-specific tool that may help clinicians diagnose pediatric AL more accurately and noninvasively.

This study has several limitations. First, it was retrospective in design, with an inherent risk of selection bias and incomplete data. Second, the control group included children with infectious diseases rather than healthy individuals; however, performing FDG-PET/CT in healthy children without clinical indication is ethically unacceptable. Inflammatory or autoimmune diseases have also been shown to elevate bone marrow FDG uptake [7,8,9], suggesting that our control group is appropriate. Third, this study does not establish FDG-PET/CT as an independent diagnostic tool for acute leukemia. Pediatric acute leukemia is classified into multiple risk groups based on cytogenetic and molecular characteristics, and bone marrow aspiration/biopsy remains the gold standard for definitive diagnosis. Rather, we emphasize that FDG-PET/CT may provide supportive information in situations where bone marrow examination is inconclusive or technically challenging (e.g., dry tap on bone marrow aspiration), and that striking bone marrow uptake on systemic FDG-PET/CT should prompt consideration of acute leukemia in the differential diagnosis. Fourth, we did not include other pediatric malignancies or hematologic conditions such as severe anemia. Alam et al. [14] reported that high bone marrow uptake may also occur in leukemia and lymphoma. Although the primary focus of disease may lie outside the bone marrow, malignancies with associated bone marrow infiltration could show FDG accumulation in the bone marrow comparable to that observed in acute leukemia. We have emphasized that further studies including a broader spectrum of pediatric malignancies are warranted to clarify disease-specific patterns. Finally, the sample size was limited. Large-scale, prospective, multicenter studies and external validation with independent datasets are needed to confirm these findings and establish the diagnostic utility of this scoring system.

## 5. Conclusions

FDG-PET/CT in children with AL demonstrated high FDG accumulation in bone marrow across the body. A simple scoring system assessing FDG uptake in the mid-diaphyseal regions of the extremities and the extent of bone marrow involvement proved as useful in distinguishing AL from IDs as using the SUV max. FDG-PET/CT-based visual scoring may provide supportive information alongside conventional diagnostics in pediatric acute leukemia.

## Figures and Tables

**Table 1:** The clinical characteristics and the blood tests between the AL and ID groups.

	ID (Range)	AL (Range)	*p*-Value
Median age (years)	6.5 (0.6–16)	7 (2–17)	0.74
Sex (boys; girls)	13; 8	4; 2	0.83
WBC (/μL)	6135 (1910–30,670)	9870 (2550–19,000)	0.11
Hb (g/dL)	12.8 (8.9–15.8)	8.8 (3.5–13.1)	<0.01
Plt (×10^4^/μL)	24.1 (2.8–62.2)	10.4 (1.6–34.0)	0.03
CRP (mg/dL) *	0.28 (0.03–29.3)	1.15 (0.01–3.52)	0.89
Ferritin (ng/mL)	208 (20.5–653)	270 (111–565)	0.64
sIL-2R (U/mL)	863 (707–1011)	1855 (488–22,869)	0.825
ESR (mm/hr)	31 (6–111)	140	0.147

* There were changes in case number (ID to AL): CRP (21:5), ferritin (7:5), IL-2 (9:4), and ESR (11:1).

## Data Availability

Data are available upon request.

## Supplementary Materials

The following supporting information can be downloaded at: https://www.mdpi.com/article/10.3390/children12091218/s1, Table S1: Characteristics of the Acute Leukemia Group. Table S2: The maximum standardized uptake value (SUVmax) values for all cases. Table S3: The visual scores and spread scores for all cases.

## Abbreviations

The following abbreviations are used in this manuscript:

FDG-PET/CT	Fluorine-18 fluorodeoxyglucose positron emission tomography/computed tomography
FUO	fever of unknown origin
AL	acute leukemia
ID	inflammatory disease
SUV max	maximum standardized uptake value
VS	visual score
SS	spread score
ROC	receiver operating characteristic
AUC	area under the curve
NCGM	National Center for Global Health and Medicine
MIP	maximum intensity projection
Hb	Hemoglobin
Plt	Platelet
WBC	White blood cell
CRP	C-reactive protein
sIL-2R	soluble interleukin-2 receptor
ESR	erythrocyte sedimentation rate
